# Supplementary material for: A vaccine antigen central in influenza A(H5) virus antigenic space confers subtype-wide immunity
Source: bioRxiv. 2024 Aug 6:2024.08.06.606696. Preprint. [Version 1] doi: 10.1101/2024.08.06.606696 (PMC11566024; doi:10.1101/2024.08.06.606696)
Supplement: Supplement 5 [file media-5.zip › Data_S2.html]

Data S2


Data S2

## Row

####

## Row

## Row

**Data S2.Three-dimensional A(H5) influenza antigenic
map.** An interactive version of the three-dimensional antigenic
map constructed from the final 117x29 dataset, shown in Fig. 1B.
Antigens are displayed as closed spheres, and sera are displayed as open
cubes. Antigens and sera are color-coded based on the genetic HA clade,
as indicated on the right-hand side of the figure. Antigens and sera
names can be visualized by hovering over the points. Each direction (x,
y, z) represents antigenic distance, and one square of the grid
corresponds to one antigenic unit, which is defined as a two-fold
difference in HI titer. The antigenic map can be rotated by clicking and
dragging in the panel. On the top right are different functions to
explore the map, and a brief description of each function appears when
hovering over. The total map stress, mean stress per titer and mean
stress per detectable titer are indicated at the bottom left.
