# Supplementary material for: A vaccine antigen central in influenza A(H5) virus antigenic space confers subtype-wide immunity
Source: bioRxiv. 2024 Aug 6:2024.08.06.606696. Preprint. [Version 1] doi: 10.1101/2024.08.06.606696 (PMC11566024; doi:10.1101/2024.08.06.606696)
Supplement: Supplement 6 [file media-6.zip › Data_S3.html]

Data S3


Data S3

## Row

### A. Piecewise procrustes 3D to 4D, 1 piece

### B. Piecewise procrustes 3D to 4D, 2 pieces

### C. Stress blobs

## Row

### D. Alternative map conformation

### E. Comparison of map conformations

### F. Bootstrap volume radii

## Row

**Data S3. Validation of the A(H5) antigenic map.**
Interactive versions of the three-dimensional antigenic map, represented
as described for Data S2. (**A**, **B**)
Piecewise Procrustes analysis (see fig. S2 and detailed in the
supplementary text) comparing the antigenic maps in three and four
dimensions. The results of analysis with one (A) and two (B) pieces are
displayed. The antigen color hue indicated which piece it belongs to,
and the shading indicates the Procrustes distance according to the
gradient displayed on the right, in antigenic units (AU).
(**C**) Triangulation blobs indicating the area in which
each datapoint can be located in the antigenic map without increasing
the total map stress by more than one unit. (**D**)
Alternative antigenic map conformation found upon comparing all 1000
optimizations (see fig. S4 and supplementary text 3). The map from
optimization 602 is shown. (**E**) The lowest stress
antigenic map (optimization 1), with Procrustes arrows pointing towards
the positions of each antigen and serum in the optimization 602 map
conformation. (**F**) Bayesian bootstrap blob size
analysis. The antigen color corresponds to the radius (AU) of a sphere
of equal volume than each blob as displayed on the right. For
interpretation, 1-2 AU differences correspond to the assay
variation.
