# Supplementary material for: A vaccine antigen central in influenza A(H5) virus antigenic space confers subtype-wide immunity
Source: bioRxiv. 2024 Aug 6:2024.08.06.606696. Preprint. [Version 1] doi: 10.1101/2024.08.06.606696 (PMC11566024; doi:10.1101/2024.08.06.606696)
Supplement: Supplement 8 [file media-8.zip › Data_S5.html]

Data S5


Data S5

## Row

### A.

### B.

## Row

**Data S5. A(H5) antigenic maps highlighting WHO candidate
virus vaccines and antigens used in the ferret vaccination
studies.** Interactive versions of the antigenic map (117x29),
represented as described for Data S2. Sera are not shown, and antigens
of interest are highlighted as opaque spheres (**A**)
Highlighting the WHO candidate virus vaccines (larger spheres) and the
WHO CVV-like (smaller spheres) antigens (see table S2).
(**B**) Highlighting antigens used in the
vaccination-challenge studies highlighted as larger spheres. The
antigenic maps can be rotated by clicking and dragging in the panel, and
scrolling allows zooming in and out.
