# Supplementary material for: A vaccine antigen central in influenza A(H5) virus antigenic space confers subtype-wide immunity
Source: bioRxiv. 2024 Aug 6:2024.08.06.606696. Preprint. [Version 1] doi: 10.1101/2024.08.06.606696 (PMC11566024; doi:10.1101/2024.08.06.606696)
Supplement: Supplement 9 [file media-9.zip › Data_S6.html]

Data S6


Data S6

## Row

### A. IraqVACC

3.56 AU to center | GMT: 21 | 50 detectable titers

### B. VC-VietnamVACC

2.60 AU to center | GMT: 32 | 78 detectable titers

## Row

### C. VC-IndonesiaVACC

0.80 AU to center | GMT: 36 | 80 detectable titers

### D. VC-AnhuiVACC

1.66 AU to center | GMT: 43 | 82 detectable titers

## Row

**Data S6. Merged antibody profiles upon vaccination with
whole-inactivated vaccines containing engineered HA antigens.**
An interactive version of the antibody profiles displayed in Fig. 2. For
each HA vaccine antigen, the position, breadth, and height of a mean
merged serum per group (n=6) are represented in the antigenic map from
Fig. 1B. HA present in vaccine: (**A**)
IraqVACC, (**B**) CVA-VietnamVACC,
(**C**) CVA-IndonesiaVACC, (**D**)
CVA-AnhuiVACC. Representation is as described for Fig. 2. In
addition, the map orientation can be changed by clicking and dragging
within the visualization, and scrolling allows zooming in and out.
Antigen names can be visualized by hovering over the points. GMT:
geometric mean titer.
