# Supplementary material for: A vaccine antigen central in influenza A(H5) virus antigenic space confers subtype-wide immunity
Source: bioRxiv. 2024 Aug 6:2024.08.06.606696. Preprint. [Version 1] doi: 10.1101/2024.08.06.606696 (PMC11566024; doi:10.1101/2024.08.06.606696)
Supplement: Supplement 10 [file media-10.zip › Data_S7.html]

Data S7


Data S7

## Row

### A. IraqVACC, I

### B. IraqVACC, II

### C. VC-VietnamVACC, I

## Row

### D. VC-VietnamVACC, II

### E. VC-IndonesiaVACC, I

### F. VC-IndonesiaVACC, II

## Row

**Data S7. Individual antibody profiles upon vaccination with
whole-inactivated vaccines containing engineered HA antigens.** 
Individual animal data used to generate merged antibody profiles
displayed in Fig. 2 and Data S6. For each HA vaccine antigen, the
position, breadth, and height of individual sera are represented in the
antigenic map from Fig. 1B. HA present in vaccine: (**A**,
**B**) IraqVACC, (**C**,
**D**) CVA-VietnamVACC, and (**E**,
**F**) CVA-IndonesiaVACC. Using the same
representation as Data S6. GMT: geometric mean titer.
