# Supplementary material for: A vaccine antigen central in influenza A(H5) virus antigenic space confers subtype-wide immunity
Source: bioRxiv. 2024 Aug 6:2024.08.06.606696. Preprint. [Version 1] doi: 10.1101/2024.08.06.606696 (PMC11566024; doi:10.1101/2024.08.06.606696)
Supplement: Supplement 11 [file media-11.zip › Data_S8.html]

Data S8


Data S8

## Row

### A. Giza challenge, AnhuiVACC

3.51 AU to center | GMT: 9 | 27 detectable titers

### B. Giza challenge, AC-AnhuiVACC

2.27 AU to center | GMT: 27 | 69 detectable titers

### C. Giza challenge, GizaVACC

2.78 AU to center | GMT: 16 | 48 detectable titers

## Row

### D. Sichuan challenge, AnhuiVACC

3.60 AU to center | GMT: 6 | 7 detectable titers

### E. Sichuan challenge, AC-AnhuiVACC

1.73 AU to center | GMT: 10 | 38 detectable titers

### F. Sichuan challenge, SichuanVACC

4.23 AU to center | GMT: 7 | 21 detectable titers

## Row

**Data S8. Merged antibody profiles of upon vaccination with
split-inactivated vaccines containing wild-type HA antigens or the
antigenically central HA antigen.**  An interactive version of
the antibody profiles displayed in Fig. 3. For each group, the position,
breadth and height of a mean merged serum per group (n=6) are
represented in the antigenic map from Fig. 1B.
(**A**-**C**) Immune responses upon
vaccination with A(H5N6) split-inactivated vaccines in the Giza
challenge study or (**D**-**F**) A(H5N1)
split-inactivated vaccines in the Sichuan challenge study. HA antigen
present in vaccine: (A, D) AnhuiVACC, (B, E)
AC-AnhuiVACC, (C) GizaVACC, and (F)
SichuanVACC. Using the same representation as Data S6. GMT:
geometric mean titer.
