# Supplementary material for: A vaccine antigen central in influenza A(H5) virus antigenic space confers subtype-wide immunity
Source: bioRxiv. 2024 Aug 6:2024.08.06.606696. Preprint. [Version 1] doi: 10.1101/2024.08.06.606696 (PMC11566024; doi:10.1101/2024.08.06.606696)
Supplement: Supplement 12 [file media-12.zip › Data_S9.html]

Data S9


Data S9

## Row

### A. Giza challenge, AnhuiVACC, I

### B. Giza challenge, AnhuiVACC, II

### C. Giza challenge, AnhuiVACC, III

### D. Giza challenge, AnhuiVACC, IV

### E. Giza challenge, AnhuiVACC, V

### F. Giza challenge, AnhuiVACC, VI

## Row

### G. Giza challenge, AC-AnhuiVACC, I

### H. Giza challenge, AC-AnhuiVACC, II

### I. Giza challenge, AC-AnhuiVACC, III

### J. Giza challenge, AC-AnhuiVACC, IV

### K. Giza challenge, AC-AnhuiVACC, V

### L. Giza challenge, AC-AnhuiVACC, VI

## Row

### M. Giza challenge, GizaVACC, I

### N. Giza challenge, GizaVACC, II

### O. Giza challenge, GizaVACC, III

### P. Giza challenge, GizaVACC, IV

### Q. Giza challenge, GizaVACC, V

### R. Giza challenge, GizaVACC, VI

## Row

**Data S9. Individual antibody profiles of animals from the
Giza vaccination-challenge study.** Individual immune responses
upon vaccination with A(H5N6) split-inactivated vaccines in the Giza
challenge study. Individual animal data used to generate merged antibody
profiles displayed in Fig. 3 and Data S8. For each HA vaccine antigen,
the position, breadth, and height of individual sera are represented in
the antigenic map from Fig. 1B. HA antigen present in vaccine:
(**A**-**F**) AnhuiVACC,
(**G**-**L**) AC-AnhuiVACC, and
(**M**-**R**) GizaVACC. Using the
same representation as Data S6. GMT: geometric mean titer.
