# Supplementary material for: A vaccine antigen central in influenza A(H5) virus antigenic space confers subtype-wide immunity
Source: bioRxiv. 2024 Aug 6:2024.08.06.606696. Preprint. [Version 1] doi: 10.1101/2024.08.06.606696 (PMC11566024; doi:10.1101/2024.08.06.606696)
Supplement: Supplement 13 [file media-13.zip › Data_S10.html]

Data S10


Data S10

## Row

### A. Sichuan challenge, AnhuiVACC, I

### B. Sichuan challenge, AnhuiVACC, II

### C. Sichuan challenge, AnhuiVACC, III

### D. Sichuan challenge, AnhuiVACC, IV

### E. Sichuan challenge, AnhuiVACC, V

### F. Sichuan challenge, AnhuiVACC, VI

## Row

### G. Sichuan challenge, AC-AnhuiVACC, I

### H. Sichuan challenge, AC-AnhuiVACC, II

### I. Sichuan challenge, AC-AnhuiVACC, III

### J. Sichuan challenge, AC-AnhuiVACC, IV

### K. Sichuan challenge, AC-AnhuiVACC, V

### L. Sichuan challenge, AC-AnhuiVACC, VI

## Row

### M. Sichuan challenge, SichuanVACC, I

### N. Sichuan challenge, SichuanVACC, II

### O. Sichuan challenge, SichuanVACC, III

### P. Sichuan challenge, SichuanVACC, IV

### Q. Sichuan challenge, SichuanVACC, V

### R. Sichuan challenge, SichuanVACC, VI

## Row

**Data S10. Individual antibody profiles of animals from the
Sichuan vaccination-challenge study.** Individual immune
responses upon vaccination with A(H5N1) split-inactivated vaccines in
the Sichuan challenge study. Individual animal data used to generate the
merged antibody profiles displayed in Fig. 3 and Data S8. For each HA
vaccine antigen, the position, breadth, and height of individual sera
are represented in the antigenic map from Fig. 1B. HA antigen present in
vaccine: (**A**-**F**) AnhuiVACC,
(**G**-**L**) AC-AnhuiVACC, and
(**M**-**R**) SichuanVACC. Using
the same representation as Data S6. GMT: geometric mean titer.
